# Sleep duration and the risk of cancer: a systematic review and meta-analysis including dose–response relationship

**DOI:** 10.1186/s12885-018-5025-y

**Published:** 2018-11-21

**Authors:** Yuheng Chen, Fengwei Tan, Luopei Wei, Xin Li, Zhangyan Lyu, Xiaoshuang Feng, Yan Wen, Lanwei Guo, Jie He, Min Dai, Ni Li

**Affiliations:** 1 Cancer Foundation of China, National Cancer Center/National Clinical Research Center for Cancer/Cancer Hospital, Chinese Academy of Medical Sciences and Peking Union Medical College, Beijing, 100021 China; 2 Department of Thoracic Surgery, National Cancer Center/National Clinical Research Center for Cancer/Cancer Hospital, Chinese Academy of Medical Sciences and Peking Union Medical College, Beijing, 100021 China; 3 Office for Cancer Early Diagnosis and Treatment, National Cancer Center/National Clinical Research Center for Cancer/Cancer Hospital, Chinese Academy of Medical Sciences and Peking Union Medical College, Beijing, 100021 China; 4 Henan Office for Cancer Control and Research, the Affiliated Cancer Hospital of Zhengzhou University, Henan Cancer Hospital, Zhengzhou, 450008 China

**Keywords:** Cancer incidence, Sleep duration, Categorical meta-analysis, Dose–response meta-analysis

## Abstract

**Background:**

The effect of sleep duration on cancer risk remains controversial. We aimed to quantify the available evidence on this relationship using categorical and dose–response meta-analyses.

**Methods:**

Population-based cohort studies and case-control studies with at least three categories of sleep duration were identified by searching PubMed, EMBASE, and the Cochrane Library database up to July 2017.

**Results:**

Sixty-five studies from 25 articles were included, involving 1,550,524 participants and 86,201 cancer cases. The categorical meta-analysis revealed that neither short nor long sleep duration was associated with increased cancer risk (short: odds ratio [OR] = 1.01, 95% confidence intervals [CI] = 0.97–1.05; long: OR = 1.02, 95% CI = 0.97–1.07). Subgroup analysis revealed that short sleep duration was associated with cancer risk among Asians (OR = 1.36; 95% CI: 1.02–1.80) and long sleep duration significantly increased the risk of colorectal cancer (OR = 1.21; 95% CI: 1.08–1.34). The dose–response meta-analysis showed no significant relationship between sleep duration and cancer risk. When treated as two linear piecewise functions with a cut point of 7 h, similar nonsignificant associations were found (per 1-h reduction: OR = 1.02, 95% CI = 0.98–1.07; per 1-h increment: OR = 1.003, 95% CI = 0.97–1.03).

**Conclusion:**

Categorical meta-analysis indicated that short sleep duration increased cancer risk in Asians and long sleep duration increased the risk of colorectal cancer, but these findings were not consistent in the dose–response meta-analysis. Long-term randomized controlled trials and well-designed prospective studies are needed to establish causality and to elucidate the mechanism underlying the association between sleep duration and cancer risk.

**Electronic supplementary material:**

The online version of this article (10.1186/s12885-018-5025-y) contains supplementary material, which is available to authorized users.

## Background

Cancer is a major public health problem worldwide and a leading cause of death in both more and less economically developed countries; the global burden of cancer is expected to increase because of population growth and aging [1]. Some lifestyle behaviors, such as smoking, alcohol consumption, weight gain, physical inactivity, and delayed or foregone reproduction (e.g., lower parity or later age at first birth) increase cancer risk [2–7].

Sleep is an essential component of healthy development and necessary for physical and mental health [8]. Increased attention has been paid to understanding the extent of sleep duration problems at the population level and the associations between these problems and various health outcomes, such as cancer, metabolic syndrome, diabetes mellitus, and all-cause mortality [9–12]. Previous studies indicate that the prevalence of short sleep duration (< 7 h) may have gradually increased over past decades, whereas the prevalence of long sleep duration (≥9 h) has decreased [13]. Epidemiological studies are a valuable way of exploring relationships between sleep and health in the general population [14]. These studies measure sleep and related health variables at a population level, elucidating relationships that may be too subtle to detect in laboratory studies but that are nonetheless useful to society [15]. Epidemiological evidence on the association between sleep duration and cancer risk is controversial, with findings showing inverse [16–18], positive [17, 19–21], and null [22–24] effects. In addition, the dose–response relationship for different quantitative categories of sleep duration in previous studies is unclear [16–18, 24].

The objective of this meta-analysis was to update the evidence on the relationship between sleep duration and cancer risk. We also aimed to explore the quantitative estimates, determine the overall shape of the relationships between sleep duration and cancer incidence, and compare categorical and dose–response meta-analyses.

## Methods

### Data sources and searches

The systematic review and meta-analyses were conducted in accordance with the Preferred Reporting Items for Systematic Reviews and Meta-analyses (PRISMA) Guidelines [25, 26]. We comprehensively identified studies through searching PubMed, EMBASE, and the Cochrane Library database up to July 2017 for both cohort and case-control studies that assessed the association between sleep duration and cancer risk. The following key words were used in the search strategy: (sleep or sleep duration) and (cancer or carcinoma or tumor). The reference lists of retrieved articles were also scanned to locate additional relevant studies.

### Study selection criteria

One investigator (YHC) conducted the initial screening of the article titles and abstracts identified in the first screening process. Two investigators (YHC) and (FWT) independently reviewed the full text of the potentially relevant articles for final inclusion, and any disagreement was resolved through discussion.

For inclusion, studies had to meet the following criteria: (1) original article; (2) cohort study, case-control study, or nested case-control study; (3) estimates and 95% confidence intervals (CI) (or the raw data to calculate these) for the association between sleep duration and the incident risk of single common cancer or multiple cancers reported in the literature; (4) adult population; and (5) published in English. Studies were excluded if they (1) had duplicated data; (2) were reviews, reports, clinical trials, or genetic and cell studies; or (3) had insufficient data. If more than one article reported data from a single study, the most recent and complete article was included.

### Data extraction and quality assessment

Data were extracted by YHC and independently checked by FWT for accuracy and completeness. Any disagreements were resolved by discussion. For each study, we extracted data on study design; first author’s surname; publication year; country of study; study name (cohort studies only); study period; gender of subjects; sample size; types of cancer; number of cases; reference category for sleep; categorization of “short” and “long” sleep duration; fully adjusted relative risks (RRs), odds ratios (ORs), or hazard ratios (HRs) for the associations of both short and long sleep duration with cancer risk; corresponding 95% confidence intervals (95% CIs); and the covariates adjusted in the statistical analysis.

Two investigators independently evaluated the quality of the included cohort and case-control studies using the Newcastle–Ottawa Scale (NOS). The selection, comparability, and exposure of each study were broadly assessed and studies were assigned a score from zero to nine. Studies with scores ≥7 were considered of high quality.

### Statistical analysis

We performed categorical and dose–response meta-analyses [27]. Random-effects models were used to pool risk estimates. The adjusted RRs, HRs, ORs, and corresponding 95% CIs were extracted from the selected studies and used to evaluate the association between sleep duration and the incident risk of any type of cancer. In this meta-analysis, the OR was deemed equivalent to RR and HR, as cancer is a rare outcome [28].

The categorical meta-analysis was conducted by pooling basic classification results of cancer incidence at different levels of sleep duration. In the original articles, sleep duration was assessed using self-report questionnaire measures of habitual sleep duration. We differentiated three levels of sleep duration: short, medium, and long. Short sleep duration was defined as follows in the different articles (in hours per night): < 5 h [17, 29], ≤5 h [18, 20, 21, 30–32], < 6 h [22, 33–36], ≤6 h [16, 23, 37–44], ≤6.5 h [19], and 3–6 h [24]. Long sleep duration was defined as > 7 h [35], > 8 h [36], ≥8 h [19, 37], ≥9 h [16, 17, 20–23, 29–34, 38–44], and ≥ 10 h [18, 24] of sleep per night. Medium sleep duration (the reference category) was classified in the studies as 7 h [16, 20, 21, 23, 31, 32, 37, 39, 41, 44], 8 h [18, 22, 30], 7–8 h [17, 34, 36, 38, 40, 42], 7–9 h [24], 7–7.5 h [19], 6–7 h [35], 6.1–8.9 h [43], and 7–7.9 h [29] of sleep per night. For the four studies [33, 35, 39, 44] in which the lowest (≤6 h or < 6 h) or the highest (8–9 h) sleep duration level was used as the reference category, we changed the reference group to the medium group (7 h, 6–7 h, or 7–8 h), and the RR/OR and the upper and lower CI were calculated for inclusion. Compared with the reference category, the pooled OR and 95% CI of cancer risk for both short and long sleep durations were calculated.

Studies with at least three quantitative categories of short or long sleep duration were also included in dose–response analyses. Potential nonlinear dose–response relationships between sleep duration and cancer risk were examined using a restricted cubic splines model with three knots at the 10th, 50th, and 90th percentiles of the distribution. We assigned the median or mean sleep duration in each category to the corresponding OR for each study. If the mean or median duration per category was not reported, the midpoint of the upper and lower boundaries in each category was assigned. For the open-ended risk factor classes (e.g., < 5 or > 10), we assigned a value following the algorithms suggested by Il’yasova et al. [45], choosing those algorithms that yielded the most plausible results for sleep duration. For upper open-ended categories, we assigned the value of the lower bound plus the width of the previous (second-to-highest) interval. For lower open-ended categories, we assigned the value of the upper bound minus half the width of the next (second-to-lowest) interval [46]. *P*_nonlinearity_ was identified by testing the null hypothesis that the estimated value of the second spline was equal to zero. If the null hypothesis did not hold, we conducted a linear dose–response meta-analysis to test the cancer risk associated with each additional hour of sleep. Otherwise, a nonlinear dose–response meta-analysis was conducted to identify the cancer risk associated with each hour. For linearity, if a U-, J-, or S-shape curve or a significant nonlinear shape association was observed, we treated the slope as two piecewise linear functions with the cut point of 7 h to show the separate linear trends [47]. All pooled outcome measures were determined using random-effects models, as described by DerSimonian and Laird, as these models produce more conservative results than fixed-effects models.

Heterogeneity among studies was estimated using Cochran’s Q test (reported with a *x*^2^-value and *P*-value) and the *I*^*2*^ statistic [48, 49]. For the Q test, a *P*-value of less than 0.1 was considered to indicate the presence of heterogeneity. The *I*^*2*^ statistic was used to test whether the proportion of total variation in the estimates could be explained by heterogeneity rather than chance. *I*^*2*^ values of 25, 50, and 75% were considered evidence of low, moderate, and high heterogeneity, respectively.

Subgroup analyses were carried out by study region (America, Asia, and Europe), gender (women and men), the definition of short or long sleep duration (“≤ 6 or ≤ 5” and “≥ 9 or ≥ 10”), the definition of the reference category (7 h–8 h or 7 h), the definition of short or long sleep duration versus the reference category (“≤6 vs.7” and “≥9 vs. 7”), study quality score (≥7 and < 7) and cancer type to minimize heterogeneity among the included studies. In addition, we estimated the risk of sex hormone-related cancer (including breast cancer, endometrial cancer, ovarian cancer, and prostate cancer, which are associated with sex hormone regulation) [50, 51]. In each specific population, the effects of short or long sleep duration on cancer risk were evaluated. We also conducted a sensitivity analysis by sequentially removing each individual study from the meta-analysis.

We visually inspected the symmetry of the funnel plots and performed the Begg regression test and Egger’s linear regression test to assess the possibility of publication bias [52, 53]. For funnel plot asymmetry, a contour-enhanced funnel plot of the effect size was examined to test for publication bias [54]. All statistical analyses were performed using Stata software (version 11.0; StataCorp, College Station, TX, USA). A *P*-value < 0.05 was considered statistically significant.

## Results

### Study selection

**Fig. 1:**
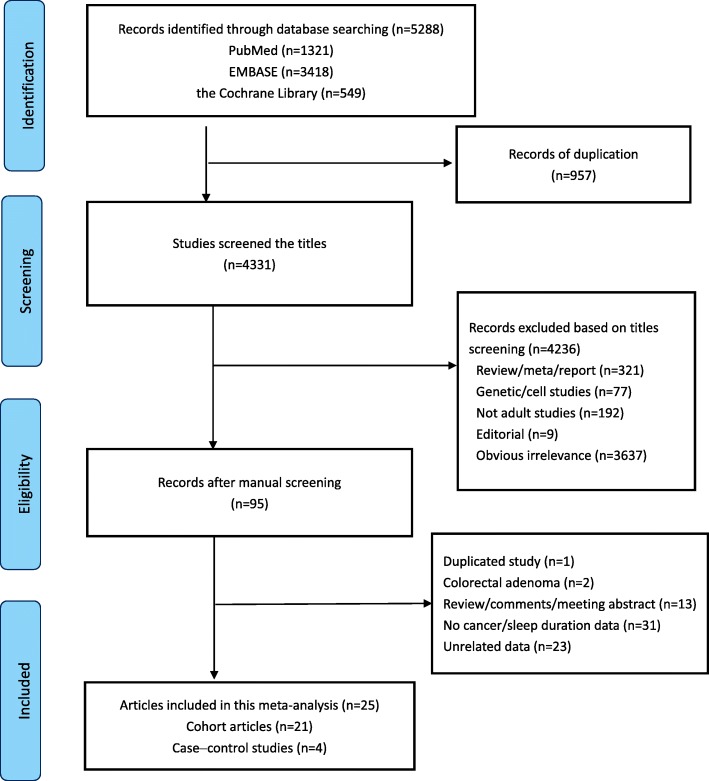
Flow chart showing the number of articles identified at each stage of the search

The literature searches identified a total of 5288 articles: 1321 from PubMed, 3418 from EMBASE, and 549 from the Cochrane Library (Fig. 1). After the initial screening of titles and the exclusion of duplicates, 95 articles were retrieved for further evaluation. The full text review showed that 1 article used a duplicated study population [55], 2 investigated the association between sleep duration and colorectal adenoma [56, 57], 13 were not original articles, and 54 were unrelated to the exposure or outcomes of interest. After excluding these articles, 25 articles that met the inclusion and exclusion criteria were used in this meta-analysis, which we performed in accordance with the guidelines of the PRISMA Statement [58] (Additional file 1).

### Study characteristics

A summary of the study characteristics is shown in Additional file 2. We identified 21 cohort articles involving 79,885 cases and 1,488,349 participants, and 4 case-control studies involving 6316 cases and 55,859 control subjects. Two of the twenty-one cohort articles reported results for three and six different types of cancer [16, 24], two articles reported the results for men and women [21, 23], and one article reported results for 15 different cancer types among men and 17 among women [17]. These were considered as separate studies in the analysis. Therefore, a total of 25 articles including 65 studies were included in the final meta-analysis. Descriptive data for the 65 included studies are summarized in Additional file 2. Twenty-two studies [17–19, 21, 23, 30, 37, 40] were conducted with men, forty-two [16, 17, 20–24, 29, 31–33, 35, 36, 38, 39, 41–44] with women, and one [34] with both men and women. Fifty-five studies were conducted in the United States [16–18, 20–24, 29, 31–33, 37–39], three in Japan [35, 40, 41], two in Finland [19, 42], one in Australia [36], one in China [43], one in Europe [34], one in Singapore [44], and one in Sweden [30]. Study size ranged from 1975 to 173,327 participants. Duration of sleep was assessed using either questionnaires or interviews that measured self-reported habitual sleep duration. All studies ascertained cancer by physician diagnosis, medical records, or cancer registry-based sources. In terms of cancer subtypes, the association between sleep duration and breast cancer, skin cancer, colorectal cancer, lung cancer, prostate cancer, endometrial cancer, ovarian cancer, thyroid cancer, other cancers and undefined cancers were reported by 12 [17, 22, 24, 29, 31–33, 36, 41–44], six [16, 23, 24], six [17, 20, 21, 24], five [17, 19, 24, 37], four [17, 18, 40], 3 [17, 24, 39], three [17, 24, 35], three [17, 38], 22 [17], and one [34] study/studies, respectively. Using the Newcastle–Ottawa Scale, the methodological quality of the studies was judged as high, with a mean score of 7.44 ± 0.65, a median of 8, a range of 6 to 8 points, and most of the studies scoring ≥7 (Additional files 3–4).

### Categorical meta-analysis

#### Short sleep duration and cancer risk

**Fig. 2:**
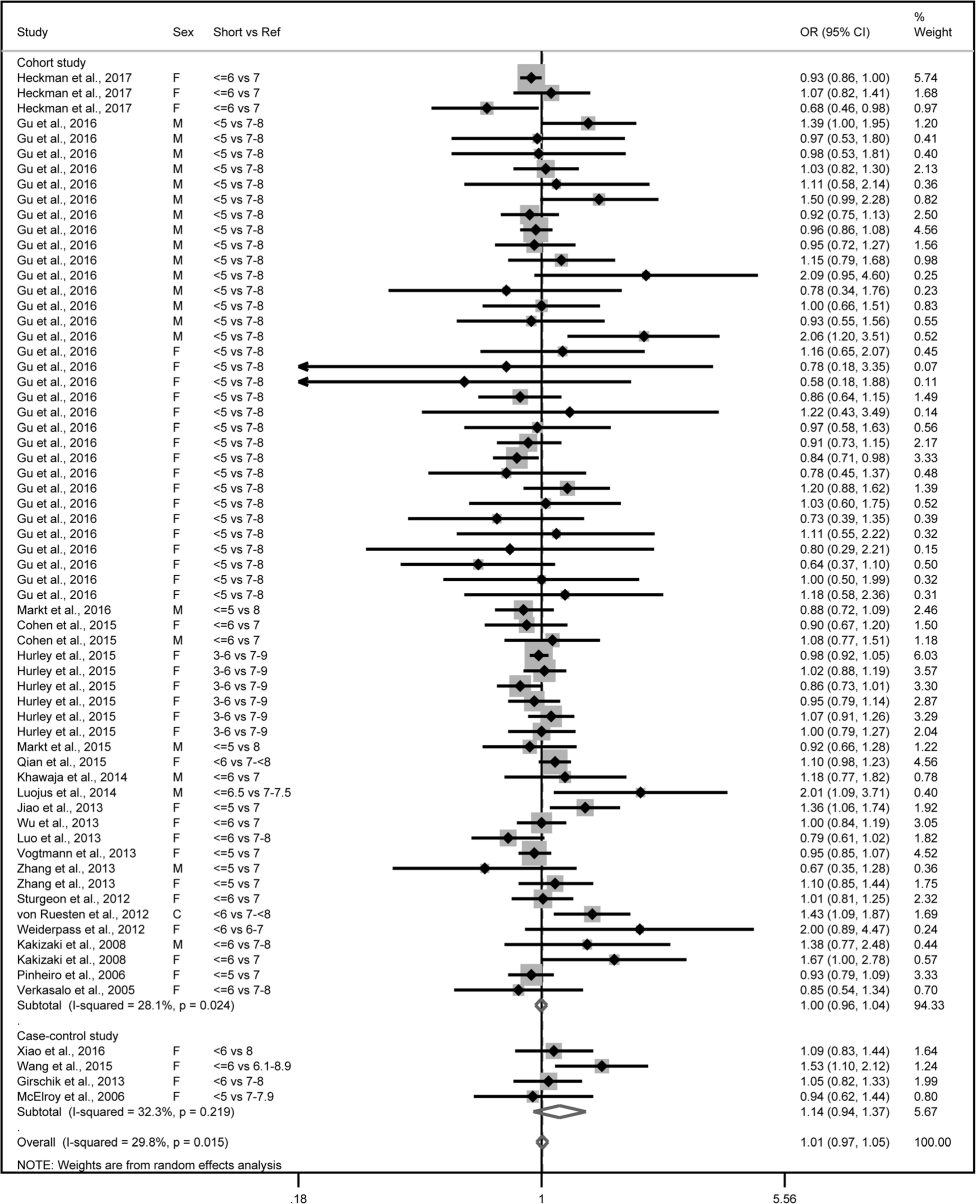
Forest plot of association between short sleep duration and cancer risk. Box sizes reflect the weights of studies included in the meta-analysis, horizontal lines are the 95% CIs, and the summary OR is represented by the diamond. OR: odds ratio, CI: confidence interval

**Fig. 3:**
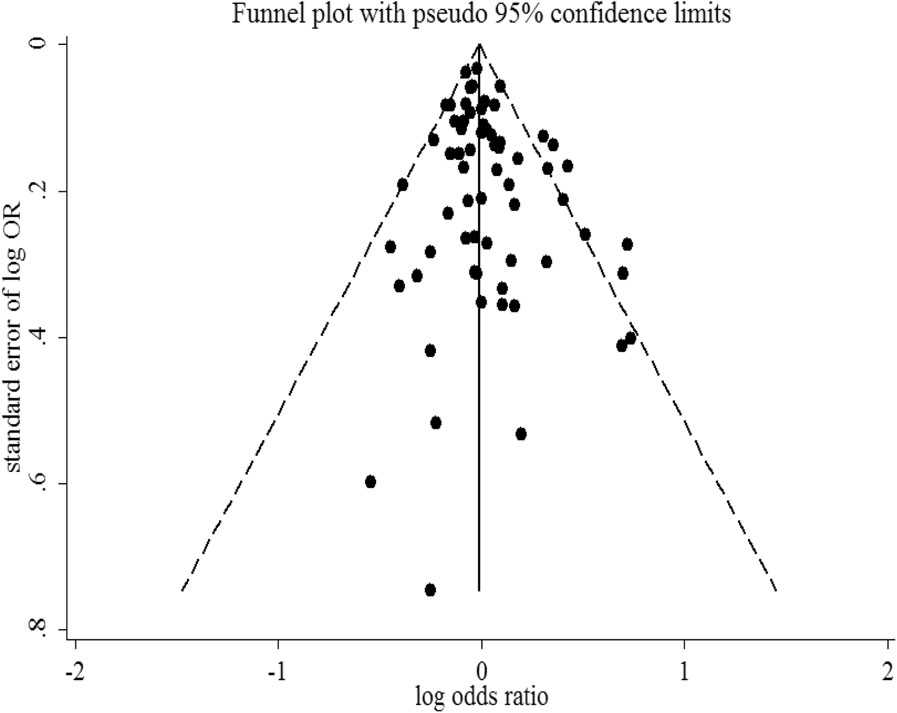
Funnel plot of studies evaluating the association between short sleep duration and cancer risk. Dotted lines indicate 95% pseudo-confidence interval. SE: standard error; OR: odds ratio

The combined OR comparing the shortest categories with the reference category of sleep duration was 1.00 (95% CI: 0.96–1.04) for the 61 cohort studies, with low to moderate heterogeneity (*P* = 0.02, *I*^2^ = 28.1%), and 1.14 (95% CI: 0.94–1.37) for the 4 case-control studies, with low to moderate heterogeneity (*P* = 0.22, *I*^2^ = 32.3%) (Fig. 2). Combining the two types of study designs resulted in an overall combined OR of 1.01 (95% CI: 0.97–1.05, *P* for heterogeneity: *P* = 0.015, *I*^*2*^ = 29.8%). Exclusion of a single study did not substantially influence the combined estimates for the cohort or case-control studies (Additional file 5). No publication bias was detected for short sleep duration and cancer risk (Egger’s test: *P* = 0.051; Begg’s test: *P* = 0.275) in the selected studies (Fig. 3). As shown in Table 1, short sleep duration was associated with cancer risk only in the Asian population (OR = 1.36; 95% CI: 1.02–1.80). Cancer risk did not vary substantially by sleep duration in most subgroups.

**Table 1:** Subgroup analyses of association between sleep duration and cancer risk

Subgroups	Short sleep duration				Long sleep duration			
	*n*	OR (95% CI)	I^2^ (%)	P_Heterogeneity_	*n*	OR (95% CI)	I^2^ (%)	P_Heterogeneity_
Regions								
USA	55	0.98 (0.95–1.02)	13.2	0.207	55	1.01 (0.97–1.06)	7.4	0.320
Asia	5	1.36 (1.02–1.80)	58.1	0.049	5	0.75 (0.44–1.27)	82.0	<0.001
Europe	4	1.19 (0.84–1.67)	66.7	0.029	4	0.95 (0.64–1.41)	70.6	0.017
Gender								
Women	42	0.98 (0.94–1.03)	22.4	0.102	42	1.02 (0.96–1.09)	30.0	0.037
Men	22	1.06 (0.96–1.16)	29.8	0.094	22	1.02 (0.94–1.11)	33.3	0.066
Definition of short or long sleep duration								
≤ 6 or ≥ 9	14	1.01 (0.92–1.11)	44.4	0.037	54	1.01 (0.95–1.06)	27.7	0.034
≤ 5 or ≥ 10	6	1.00 (0.87–1.15)	51.1	0.069	7	1.14 (0.87–1.49)	48.7	0.069
Definition of reference category								
7-8 h or 7 h	51	1.00 (0.95–1.05)	24.6	0.061	51	1.00 (0.95–1.05)	21.9	0.088
Definition of short sleep or long duration versus reference category								
≤ 6 vs. 7 or ≥ 9 vs. 7	9	0.98 (0.90–1.07)	27.0	0.204	15	0.99 (0.90–1.08)	43.3	0.038
Study quality score								
≥ 7	58	1.02 (0.97–1.07)	33.9	0.007	58	1.01 (0.95–1.06)	32.4	0.011
< 7	7	0.97 (0.93–1.02)	0.00	0.627	7	1.14 (0.95–1.37)	26.5	0.226
Cancer type								
Sex hormone-related cancers	22	0.99 (0.94–1.04)	32.9	0.069	22	0.97 (0.89–1.06)	51.0	0.003
Breast cancer	12	1.00 (0.94–1.08)	46.1	0.040	12	1.02 (0.92–1.12)	51.0	0.021
Skin cancer	6	0.93 (0.88–1.00)	0.0	0.481	6	0.92 (0.78–1.10)	19.4	0.287
Colorectal cancer	6	1.05 (0.92–1.19)	38.3	0.151	6	1.21 (1.08–1.34)	0.0	0.555
Endometrial cancer	3	0.98 (0.82–1.17)	44.3	0.135	3	1.06 (0.83–1.34)	0.0	0.589
Lung cancer	5	1.04 (0.88–1.22)	46.1	0.115	5	1.01 (0.83–1.23)	41.6	0.144
Ovarian cancer	3	1.05 (0.72–1.53)	44.3	0.166	3	0.84 (0.46–1.52)	60.5	0.079
Prostate cancer	4	0.95 (0.86–1.04)	0.0	0.540	4	0.75 (0.54–1.05)	70.9	0.016
Thyroid cancer	3	1.11 (0.64–1.93)	65.0	0.058	3	0.95 (0.63–1.45)	0.0	0.539

Asia: China, Japan and Singapore; Europe: Europe, Finland and Sweden

Sex hormone-related cancers: included breast cancer, endometrial cancer, ovarian cancer, and prostate cancer

Skin cancer: included basal cell skin cancer, squamous cell skin cancer, and melanoma

#### Long sleep duration and cancer risk

**Fig. 4:**
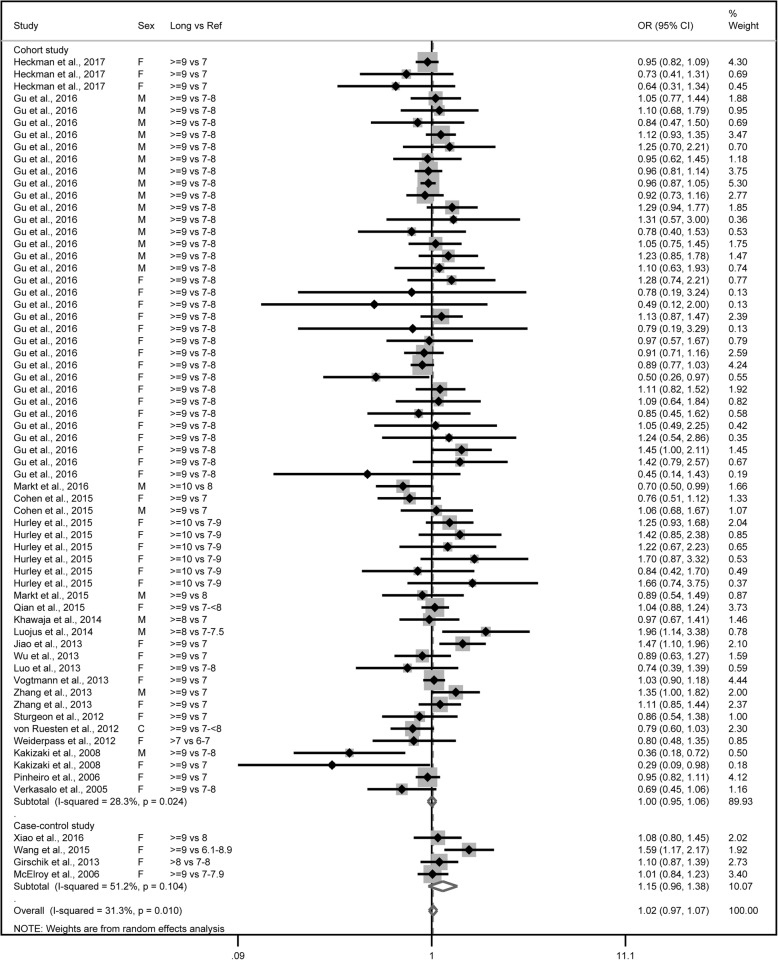
Forest plot of association between long sleep duration and cancer risk. Box sizes reflect the weights of studies included in the meta-analysis, horizontal lines are the 95% CIs, and the summary OR is represented by the diamond. OR: odds ratio, CI: confidence interval

**Fig. 5:**
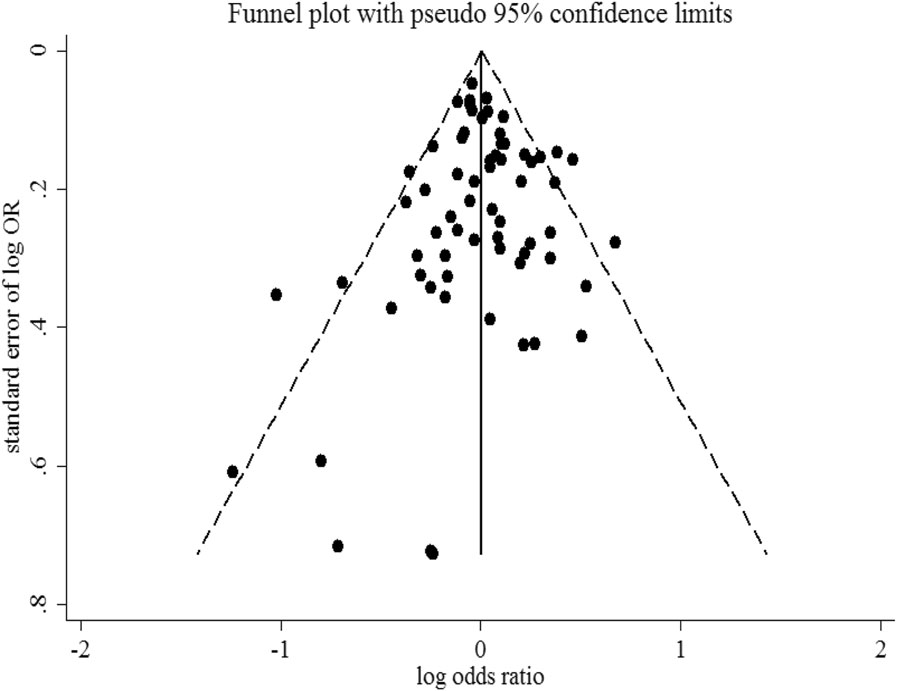
Funnel plot of studies evaluating the association between long sleep duration and cancer risk. Dotted lines indicate 95% pseudo-confidence interval. SE: standard error; OR: odds ratio

The combined OR comparing the longest sleep categories and the reference category of sleep duration was 1.00 (95% CI: 0.95–1.06) for the 61 cohort studies with low to moderate heterogeneity (*P* = 0.02, *I*^*2*^ = 28.3%) and 1.15 (95% CI: 0.96–1.38) with moderate heterogeneity (*P* = 0.10, *I*^*2*^ = 51.2%) for the 4 case-control studies (Fig. 4). Combining the two types of study designs resulted in an overall combined OR of 1.02 (95% CI: 0.97–1.07, *P* for heterogeneity: *P* = 0.01, *I*^*2*^ = 31.3%). Exclusion of a single study did not substantially influence the combined estimates for the cohort or case-control studies (Additional file 6). No publication bias was detected for long sleep duration and cancer risk (Egger’s test: *P* = 0.935; Begg’s test: *P* = 0.305) in the selected studies (Fig. 5). As shown in Table 1, in terms of cancer type, a significant association was found between long sleep duration and colorectal cancer (OR = 1.21; 95% CI: 1.08–1.34). No significant associations were observed when the data were stratified by study region, gender, or definition of sleep duration.

### Dose–response meta-analysis

**Fig. 6:**
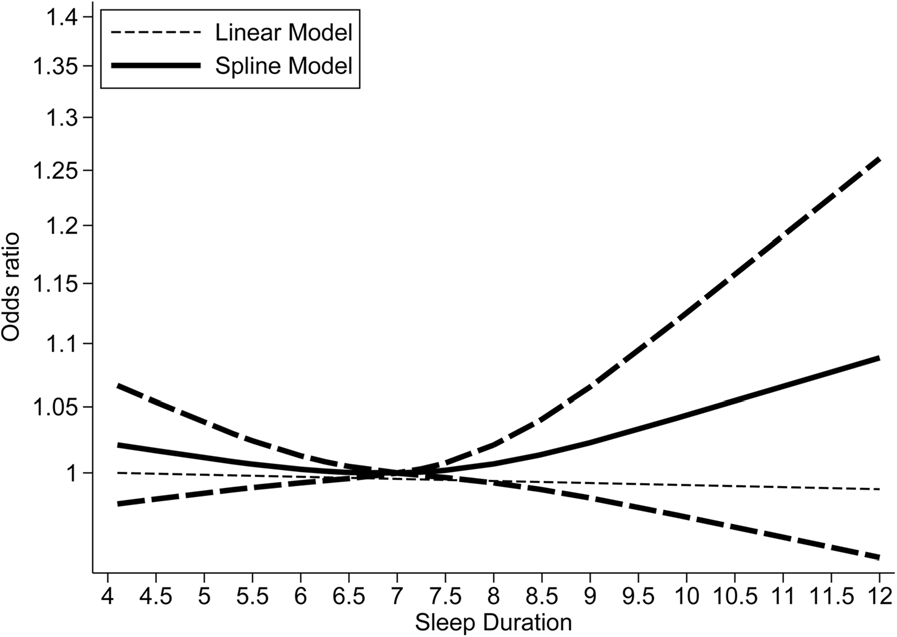
Nonlinear dose–response analyses of sleep duration and cancer risk. The solid line and the long-dashed line represent the estimate odds ratios and their 95% confidence intervals. Seven hours of sleep per night was used as the reference

Sixty-one population-based cohort studies and four case-control studies, including 86,201 cases among 1,550,524 participants, were combined in the dose–response meta-analysis of sleep duration and cancer risk. As shown in Fig. 6, the nonlinear (*P* = 0.24) dose–response analysis indicated no relationship between sleep duration and cancer risk (Additional file 7). However, a linear relationship (*P* = 0.84) was found, which suggested that increasing sleep duration (in 1-h increments) was not associated with cancer risk (OR = 0.999, 95% CI = 0.993–1.006). When treated as two piecewise linear functions, among people who slept less than 7 h per night, a 1-h reduction in sleep duration was not associated with an increase in cancer risk (OR = 1.021, 95% CI = 0.979–1.066), and the results for people with sleep durations of more than 7 h were similar (OR = 1.003, 95% CI = 0.972–1.034).

Using the available data, we examined potential effect modifications by study region, gender, study design (cohort study and case-control study), study quality score, and cancer type. The association was not appreciably modified by study region, gender, study quality, or study design. When stratified by cancer type, we observed a J-shaped curvilinear association, with the lowest colorectal cancer risk at a sleep duration of about 7 h per night (*P* = 0.005 for nonlinearity, Additional file 8). When treated as two piecewise linear functions, for people who slept for less than 7 h per night, a 1-h sleep duration reduction was not associated with colorectal cancer risk (OR = 0.994, 95% CI = 0.889–1.113); results for those with sleep durations longer than 7 h were similar (OR = 1.064, 95% CI = 0.978–1.157).

## Discussion

This study provided a comprehensive systematic review of the literature and quantitative estimates of the associations of short and long sleep duration with the risk of cancer in population-based observational studies. The findings showed that neither short nor long sleep duration was significantly associated with cancer risk, regardless of the examined categories in the meta-analysis or dose–response meta-analysis. Interestingly, the subgroup analysis revealed that short sleep duration was associated with increased risk of cancer in Asian populations (this finding was based on four cohort and one case-control study including 1779 incident cases) and long sleep duration was associated with increased risk of colorectal cancer (this finding was based on six cohort studies including 8099 incident cases).

### Comparison with previous reviews and meta-analyses

Most previous reviews and meta-analyses of studies on sleep duration and cancer risk have reported null findings or shown that longer sleep duration might be a risk factor for colorectal cancer, which is consistent with our findings [59–61]. A meta-analysis published in 2013 found that neither extremely short sleep duration nor long sleep duration was statistically associated with an increased risk of cancer in a categorical meta-analysis (short: HR = 1.06, 95% CI = 0.92–1.23; long: HR = 0.91, 95% CI = 0.78–1.07). The same meta-analysis, which included 13 cohort studies, found no significant dose–response relationship (*P* = 0.51) between sleep duration and cancer [61]. A meta-analysis of 10 cohort studies by Lu et al. [60] categorized sleep duration into three groups: short, moderate, and long. They concluded that neither short nor long sleep duration was statistically associated with increased risk of cancer (short: RR = 1.05, 95% CI = 0.90–1.24; long: RR = 0.92, 95% CI = 0.76–1.12) and that long sleep duration was positively associated with colorectal cancer (RR = 1.29, 95% CI = 1.09–1.52). Erren et al. [59] reported that the combined adjusted RR was 1.08 (95% CI = 1.03–1.13) for colorectal cancer and 1.11 (95% CI = 1.00–1.22) for lung cancer, but this analysis did not examine the dose–response relationships. We conducted a flexible, categorical, and dose–response meta-analysis and treated the slope as two piecewise linear functions with the cut point of 7 h to show the linear trends. Therefore, the present study might provide the most comprehensive assessment and robust evidence to date on the relationship between sleep duration and cancer risk.

### Potential mechanisms

The etiology of cancer is multifactorial; genetic, metabolic, environmental, behavioral, and social or cultural factors are major contributors. Although the exact mechanism remains unknown, there are several possible pathways that could explain the association between sleep duration and cancer risk. First, the melatonin hypothesis proposes that shorter sleep duration is associated with decreased levels of melatonin [62], and melatonin has been found to suppress the initiation phase of tumorigenesis and inhibit the proliferation of human cancer cell lines in experimental studies [14, 63]. Numerous studies have demonstrated the association of sex hormones with the development and progression of various types of cancer, including cancers of the breast, endometrium, ovary, and prostate [64–70]. Moreover, melatonin may modulate sex hormone production by interacting with estrogen-signaling pathways through different mechanisms [71, 72]. A recent dose-response meta-analysis by Yang et al. has found that an increase in urinary aMT6s of 15 ng/mg creatinine is associated with a 14% reduced risk of breast cancer, especially in post-menopausal women [73]. Therefore, melatonin might play a role in the progression of sex hormone-related cancers. Although several studies have evaluated the interrelation between melatonin and sex hormone levels, the interpretations of their findings are inconsistent [74–76]. The second possible mechanism is impaired immune function [77]. Both laboratory studies of acute sleep deprivation and observational studies of poor sleepers have reported that sleep duration changes may lead to a suppression of immune function and a shift in the balance of cytokine production [78, 79]. The third possible mechanism is disruption of circadian rhythms. Disruption of circadian physiology owing to an annual decrease in sleep duration or sleep disturbances may result in impaired glucose, reduced appetite control [14], and various gastrointestinal diseases, such as irritable bowel syndrome, gastroesophageal reflux disease, or peptic ulcer disease. In addition, circadian disruption can promote tumorigenesis in the liver and gastrointestinal tract [80]. The fourth mechanism involves metabolic pathways related to obesity, which is a risk factor for several cancers [81]. The decrease of sleep duration may be a stress response to chronic stress and unhealthy emotions. Chronic stress plays a significant role in cancer incidence, and depression is a risk factor for cancer onset and cancer progression [82, 83].

Our findings indicate that long sleep duration is an additional behavioral risk factor for colorectal cancer. The proposed mechanism is that sleep may influence cancer risk via alterations in levels of appetite-regulating hormones, such as leptin and ghrelin [84, 85], leading to increased appetite and subsequent obesity [84, 86–88]. The association between sleep duration and colorectal cancer may also be explained by comorbidities [89] and residual confounding. For instance, other mental or physiological disorders, low socioeconomic status, low levels of physical activity, and undiagnosed chronic comorbid conditions may be correlated with long sleep duration; these factors could confound the association between sleep duration and cancer incidence [61, 90].

We also performed a subgroup analysis by study population. Short sleep duration was strongly associated with increased cancer incidence in Asian participants but not in American and European participants. Several possible pathways could explain the relationship between short sleep duration and increased cancer incidence in Asians. First, there may be differences in melatonin secretion patterns between Asians and Americans [91]. Wetterberg et al. have published two studies comparing urinary melatonin in women in Asian (Japanese) and Caucasian (American) populations. Both studies found significantly lower levels of melatonin excretion in Japanese women compared with American women [92, 93]. Although it was not possible to investigate these effects in the present study, ethnic background should be considered a variable of interest in future studies. Second, differences in sleep patterns (e.g., daytime naps, the use of sleep medication, and sleeping alone or with a partner) across different countries might have affected the results [94]. In a Chinese population-based study of 452,829 adults aged 30–79 years, 20.3% of participants had daytime naps all year round and 40.1% had daytime naps in summer [95]. Napping increases sleep duration and may be correlated with sleep disturbances or poor sleep [59]. In addition, the results of our meta-analysis should be viewed with caution owing to heterogeneity caused by discrepancies in sample sizes, sample characteristics, response rates, and sleep duration measures. Additional studies are needed for reliable quantification of this association and to evaluate whether these factors contribute to region-related differences.

### Strengths and limitations

Our study addressed the limitations of previous research and had several strengths. First, this meta-analysis was based on up-to-date literature and presented the largest synthesis to date of prospective cohort studies and case-control studies with large sample sizes, which increased the statistical power to detect potential associations. Second, data for the pooled analysis were derived from fully adjusted models in the primary studies, which should reduce the likelihood of confounding. Moreover, the combined use of categorical and dose–response meta-analyses provided more information. Linear and nonlinear relationships were also tested to assess the dose–response relationship, and we performed subgroup and sensitivity analyses on potential confounders. The methodological features of our study enhance the quality of our results and strengthen the validity of the conclusions.

Several limitations of this meta-analysis should be considered. First, meta-analyses are greatly influenced by the quality of the individual studies included. Nearly all the included studies relied on self-reports of sleep duration collected via questionnaires or interviews; this type of data may not fully or accurately capture actual sleep duration. Additionally, sleep duration was classified differently across the original studies, and the differences in the reference groups were particularly large. Second, this meta-analysis summarizes results from both prospective cohort and case-control studies. However, there are many differences between these two types of study design, such as different statistical estimates (HRs, RRs, or ORs) and different biases. The previous two limitations may have led to heterogeneity in our meta-analysis. Thus, subgroup analyses, sensitivity analyses, and random-effects models were generated to examine sources of heterogeneity. Third, most of the studies assessed sleep duration at a single point in time; this method might not accurately reflect the sustained effects of sleep duration over time when relating them to the long-term development of cancer. Fourth, the available data were limited for several studies, which might have led to a loss of statistical power for some of the subgroup results. In the subgroup analysis of cancer types and study population, the categorical and dose–response analyses produced inconsistent results. It is possible that there was insufficient statistical power to detect a dose–response effect in the different groups; studies with larger subgroup sample sizes are needed to validate these associations. Fifth, most studies reported data for only breast, skin, colorectal, lung, prostate, endometrial, ovarian, and thyroid cancers, except one European study that reported data for all cancer types [34]. Therefore, the present findings cannot be generalized to all cancer types.

### Implications for practice and research

Because of limited evidence and a lack of consensus on the effects of sleep duration on cancer risk, we believe that the following aspects warrant close attention in future investigations of the association between sleep duration and cancer risk [12, 59, 96]:

1. *Study design.* Additional targeted biological research is needed to determine the exact mechanisms underlying the association between sleep duration and cancer risk, and population-based long-term epidemiological studies are needed to explore the link between specific (precisely measured) sleep durations and cancer risk.
2. *Measurement and assessment of sleep duration.* Further epidemiological studies should be conducted using electronic wearable devices to obtain precise, reliable, and scalable objective measures of sleep duration (how long), sleep timing (when), sleep quality (good or bad), and location (longitude and latitude). Such studies should also assess, as directly as possible, the effects of sleep duration and changes in sleep duration on cancer incidence and development during long-term follow-up.
3. *Confounding bias.* The presence of confounding variables is a limitation of many studies included in this meta-analysis; therefore, future studies should control for the following:
  The need to sleep with a light on (always to never);
  Sleep environmental factors (presence of young children, population density, place where the subjects sleeps, level of noise at the sleeping place);
  Sleep quality assessment (insomnia, snoring, sleep apnea, sleep deprivation);
  Sleep culture (watching TV/drinking in bed, daytime napping); and
  The use of melatonin supplementation
4. *Chronobiological variables.* Further experimental studies that focus on melatonin, hormonal rhythms, and clock gene expressions are needed to interpret the complex relationships between sleep and cancer.

## Conclusions

Our findings indicate that neither short nor long sleep duration was significantly associated with cancer risk, although short sleep duration slightly increased cancer risk among Asians and long sleep duration slightly increased the risk of colorectal cancer. Large-scale, well-designed prospective studies are required to further investigate the observed association. Long-term randomized controlled trials are needed to establish causality and to elucidate the mechanisms underlying the association between sleep duration and cancer risk.

## Additional files

Additional file 1: PRISMA 2009 Checklist. (DOCX 25 kb)

Additional file 2: Characteristics of studies included in the meta-analysis. (DOCX 39 kb)

Additional file 3: Study quality of cohort studies included in the analysis of sleep duration and cancer risk. (DOCX 21 kb)

Additional file 4: Study quality of case-control studies included in the analysis of sleep duration and cancer risk. (DOCX 20 kb)

Additional file 5: Sensitivity analysis of sleep duration and cancer risk, shortest vs. reference analysis. (PDF 194 kb)

Additional file 6: Sensitivity analysis of sleep duration and cancer risk, longest vs. reference analysis. (PDF 186 kb)

Additional file 7: Association between sleep duration and cancer risk from nonlinear dose–response analysis. (DOCX 20 kb)

Additional file 8: Nonlinear dose–response analyses of sleep duration and colorectal cancer risk. (PDF 79 kb)

## Abbreviations

95% CI: 95% confidence interval
HR: Hazard ratio
NOS: Newcastle–Ottawa Scale (NOS)
OR: Odds ratio
PRISMA: Preferred reporting items for systematic reviews and meta-analyses
RR: Relative risk
