# Supplementary material for: Sleep duration and the risk of cancer: a systematic review and meta-analysis including dose–response relationship
Source: BMC Cancer. 2018 Nov 21;18:1149. doi: 10.1186/s12885-018-5025-y (PMC6249821; doi:10.1186/s12885-018-5025-y)
Supplement: Supplementary file 2 — Characteristics of studies included in the meta-analysis. (DOCX 39 kb) [file 12885_2018_5025_MOESM2_ESM.docx]

**Additional file 2. Characteristics of studies included in the meta**–**analysis**

| Author, year, country/area | Sex^#^ | Types  of Cancer | No. of Cases | Sample sizes | Sleep  Duration | Adjusted OR/RR  /HR (95%CI) | Adjusted variable | NOS  score |
| --- | --- | --- | --- | --- | --- | --- | --- | --- |
| Cohort study | | | | | | | | |
| Heckman *et al*.,  2017, USA | F | Basal cell  skin cancer | 4308 | 74323 | ≤6  7  8  ≥9 | 0.93 (0.86–1.00)  1.00  1.03 (0.95–1.11)  0.95 (0.82–1.09) | Years of shift work, hours of sleep per night, sleep adequacy, sleep days per week, snoring, restless legs syndrome, family history of melanoma, hours spent in sun per week, number of severe sunburns from ages 15 to 20, sunburn severity after ≥2 hours in the sun during childhood, artificial tanning frequency per year ages 25-35, annual UV at residence, moles on lower legs, natural hair color in adolescence, marital status, financial status on 10-rung ladder, BMI,  physical activity, smoking status, g/day alcohol intake, oral contraceptive use, menopausal status, postmenopausal hormones, alternate healthy eating index | 7 |
|  |  | Squamous cell skin  cancer | 334 | 74323 | ≤6  7  8  ≥9 | 1.07 (0.82–1.41)  1.00  0.97 (0.73–1.28)  0.73 (0.41–1.31) |  |  |
|  |  | Melanoma | 212 | 74323 | ≤6  7  8  ≥9 | 0.68 (0.46–0.98)  1.00  1.12 (0.80–1.56)  0.64 (0.31–1.34) |  |  |
| Gu *et al*., 2016,  USA | M | Head and  neck cancer | 1044 | 173327 | <5  5–6  7–8  ≥9 | 1.39 (1.00–1.95)  1.12 (0.98–1.28)  1.00  1.05 (0.77–1.44) | Age, napping, race, education, marital status, self-reported health, family history of cancer, smoking, physical activity, sitting time, diabetes, hypertension, body mass index (BMI), NSAID use, alcohol drinking, intakes of fruits and vegetables, wholegrain, total fat, red meat, total calories | 7 |
|  |  | Esophageal cancer | 417 | 173327 | <5  5–6  7–8  ≥9 | 0.97 (0.53–1.80)  1.11 (0.90–1.37)  1.00  1.10 (0.68–1.79) |  |  |
|  |  | Stomach cancer | 409 | 173327 | <5  5–6  7–8  ≥9 | 0.98 (0.53–1.81)  1.29 (1.05–1.59)  1.00  0.84 (0.47–1.50) |  |  |
|  |  | Colorectal cancer | 2895 | 173327 | <5  5–6  7–8  ≥9 | 1.03 (0.82–1.30)  1.03 (0.94–1.11)  1.00  1.12 (0.93–1.35) |  |  |
|  |  | Liver cancer | 256 | 173327 | <5  5–6  7–8  ≥9 | 1.11 (0.58–2.14)  0.97 (0.74–1.27)  1.00  1.25 (0.70–2.21) |  |  |
|  |  | Pancreatic cancer | 657 | 173327 | <5  5–6  7–8  ≥9 | 1.50 (0.99–2.28)  0.98 (0.83–1.17)  1.00  0.95 (0.62–1.45) |  |  |
|  |  | Lung cancer | 3454 | 173327 | <5  5–6  7–8  ≥9 | 0.92 (0.75–1.13)  1.00 (0.93–1.08)  1.00  0.96 (0.81–1.14) |  |  |
|  |  | Prostate cancer | 14044 | 173327 | <5  5–6  7–8  ≥9 | 0.96 (0.86–1.08)  0.97 (0.94–1.01)  1.00  0.96 (0.87–1.05) |  |  |
|  |  | Bladder cancer | 2167 | 173327 | <5  5–6  7–8  ≥9 | 0.95 (0.72–1.27)  1.10 (1.00–1.20)  1.00  0.92 (0.73–1.16) |  |  |
|  |  | Kidney cancer | 927 | 173327 | <5  5–6  7–8  ≥9 | 1.15 (0.79–1.68)  1.03 (0.89–1.19)  1.00  1.29 (0.94–1.77) |  |  |
|  |  | Thyroid cancer | 154 | 173327 | <5  5–6  7–8  ≥9 | 2.09 (0.95–4.60)  1.16 (0.82–1.64)  1.00  1.31 (0.57–3.00) |  |  |

|  |  | Brain cancer | 322 | 173327 | <5  5–6  7–8  ≥9 | 0.78 (0.34–1.76)  0.85 (0.66–1.09)  1.00  0.78 (0.40–1.53) |  |  |
| --- | --- | --- | --- | --- | --- | --- | --- | --- |
|  |  | Non-Hodgkin  Lymphoma | 1084 | 173327 | <5  5–6  7–8  ≥9 | 1.00 (0.66–1.51)  1.17 (1.02–1.33)  1.00  1.05 (0.75–1.45) |  |  |
|  |  | Leukemia | 708 | 173327 | <5  5–6  7–8  ≥9 | 0.93 (0.55–1.56)  1.10 (0.94–1.30)  1.00  1.23 (0.85–1.78) |  |  |
|  |  | Myeloma | 349 | 173327 | <5  5–6  7–8  ≥9 | 2.06 (1.20–3.51)  1.13 (0.89–1.43)  1.00  1.10 (0.63–1.93) |  |  |
| Gu *et al*., 2016,  USA | F | Head and  neck cancer | 292 | 123858 | <5  5–6  7–8  ≥9 | 1.16 (0.65–2.07)  0.92 (0.71–1.19)  1.00  1.28 (0.74–2.21) | Age, napping, race, education, marital status, self-reported health, family history of cancer, smoking, physical activity, sitting time, diabetes, hypertension, BMI, NSAID use, alcohol drinking, intakes of fruits and vegetables, wholegrain, total fat, red meat and total calories. For breast, ovarian and endometrial cancers model additionally adjusted for postmenopausal hormonal use, menopausal status, number of live child birth, oral contraception use, hysterectomy, oophorectomy | 7 |
|  |  | Esophageal cancer | 66 | 123858 | <5  5–6  7–8  ≥9 | 0.78 (0.18–3.35)  1.22 (0.72–2.04)  1.00  0.78 (0.19–3.24) |  |  |
|  |  | Stomach cancer | 113 | 123858 | <5  5–6  7–8  ≥9 | 0.58 (0.18–1.88)  1.05 (0.7–1.55)  1.00  0.49 (0.12–2.00) |  |  |
|  |  | Colorectal cancer | 1507 | 123858 | <5  5–6  7–8  ≥9 | 0.86 (0.64–1.15) 0.99 (0.89–1.11)  1.00  1.13 (0.87–1.47) |  |  |
|  |  | Liver cancer | 83 | 123858 | <5  5–6  7–8  ≥9 | 1.22 (0.43–3.49)  1.41 (0.89–2.22)  1.00  0.79 (0.19–3.29) |  |  |
|  |  | Pancreatic cancer | 408 | 123858 | <5  5–6  7–8  ≥9 | 0.97 (0.58–1.63)  0.97 (0.78–1.2)  1.00  0.97 (0.57–1.67) |  |  |
|  |  | Lung cancer | 3454 | 123858 | <5  5–6  7–8  ≥9 | 0.91 (0.73–1.15)  1.05 (0.96–1.15)  1.00  0.91 (0.71–1.16) |  |  |
|  |  | Breast cancer | 5919 | 123858 | <5  5–6  7–8  ≥9 | 0.84 (0.71–0.98)  1.00 (0.94–1.05) 1.00  0.89 (0.77–1.03) |  |  |
|  |  | Ovarian cancer | 515 | 123858 | <5  5–6  7–8  ≥9 | 0.78 (0.45–1.37)  1.05 (0.87–1.27)  1.00  0.50 (0.26–0.97) |  |  |
|  |  | Endometrial cancer | 1030 | 123858 | <5  5–6  7–8  ≥9 | 1.20 (0.88–1.62)  0.88 (0.76–1.01)  1.00  1.11 (0.82–1.52) |  |  |
|  |  | Bladder cancer | 382 | 123858 | <5  5–6  7–8  ≥9 | 1.03 (0.60–1.75)  0.88 (0.70–1.10)  1.00  1.09 (0.64–1.84) |  |  |
|  |  | Kidney cancer | 337 | 123858 | <5  5–6  7–8  ≥9 | 0.73 (0.39–1.35)  1.06 (0.84–1.33)  1.00  0.85 (0.45–1.62) |  |  |

|  |  | Thyroid cancer | 192 | 123858 | <5  5–6  7–8  ≥9 | 1.11 (0.55–2.22)  0.87 (0.63–1.19)  1.00  1.05 (0.49–2.25) |  |  |
| --- | --- | --- | --- | --- | --- | --- | --- | --- |
|  |  | Brain cancer | 158 | 123858 | <5  5–6  7–8  ≥9 | 0.80 (0.29–2.21)  1.29 (0.92–1.79)  1.00  1.24 (0.54–2.86) |  |  |
|  |  | Non-Hodgkin Lymphoma | 628 | 123858 | <5  5–6  7–8  ≥9 | 0.64 (0.37–1.10)  0.98 (0.82–1.16)  1.00  1.45 (1.00–2.11) |  |  |
|  |  | Leukemia | 256 | 123858 | <5  5–6  7–8  ≥9 | 1.00 (0.50–1.99)  1.14 (0.87–1.49)  1.00  1.42 (0.79–2.57) |  |  |
|  |  | Myeloma | 170 | 123858 | <5  5–6  7–8  ≥9 | 1.18 (0.58–2.36)  0.83 (0.59–1.16)  1.00  0.45 (0.14–1.43) |  |  |
| Markt *et al*.,  2016, USA | M | Prostate cancer | 4261 | 32141 | ≤5  6  7  8  9  ≥10 | 0.88 (0.72–1.09)  0.93 (0.85–1.02)  1.03 (0.96–1.11)  1.00  0.88 (0.76–1.01)  0.70 (0.50–0.99) | Age, race, vigorous activity level, smoking, diabetes, family history of prostate cancer, snoring status, multivitamin use, energy intake, history of PSA testing, β-blocker use, marital use, coffee intake, alcohol intake, number of urinations per night | 8 |
| Cohen *et al*.,  2015, USA | F | Melanoma | 642 | 146704 | ≤6  7  8  ≥9 | 0.90 (0.67–1.20)  1.00  1.30 (1.08–1.56)  0.76 (0.51–1.12) | Age, number of sunburns, moles, hair color, family history of melanoma, reaction to sun, tanning, Caucasian ethnicity, ultraviolet flux, snoring | 7 |
|  | M | Melanoma | 238 | 31929 | ≤6  7  8  ≥9 | 1.08 (0.77–1.51)  1.00  0.95 (0.69–1.30)  1.06 (0.68–1.67） |  |  |
| Hurley *et al*.,  2015, USA | F | Breast cancer | 4381 | 101609 | 3–6  7–9  ≥10 | 0.98 (0.92–1.05)  1.00  1.25 (0.93–1.68) | Race, alcohol consumption, menopausal status/hormone therapy use | 6 |
|  | F | Colorectal cancer | 873 | 101609 | 3–6  7–9  ≥10 | 1.02 (0.88–1.19)  1.00  1.42 (0.85–2.38) | Race, BMI, alcohol consumption, comorbidity, menopausal status/hormone therapy use |  |
|  | F | Endometrial cancer | 821 | 101609 | 3–6  7–9  ≥10 | 0.86 (0.73–1.01)  1.00  1.22 (0.67–2.23) | Race, BMI, physical activity, comorbidity and menopausal status/hormone therapy use |  |
|  | F | Melanoma | 661 | 101609 | 3–6  7–9  ≥10 | 0.95 (0.79–1.14)  1.00  1.70 (0.87–3.32) | Race, physical activity, alcohol consumption, comorbidity, smoking status, menopausal status/hormone therapy use, neighborhood urbanization and socioeconomic status |  |
|  | F | Lung cancer | 728 | 101609 | 3–6  7–9  ≥10 | 1.07 (0.91–1.26)  1.00  0.84 (0.42–1.70) | Race, BMI, physical activity, alcohol consumption, comorbidity, smoking status, neighborhood urbanization |  |
|  | F | Ovarian cancer | 374 | 101609 | 3–6  7–9  ≥10 | 1.00 (0.79–1.27)  1.00  1.66 (0.74–3.75) | Race, BMI, menopausal status/hormone therapy use |  |
| Markt *et al*.,  2015, Sweden | M | Prostate cancer | 785 | 12976 | ≤5  6  7  8  ≥9 | 0.92 (0.66–1.28)  0.93 (0.76–1.14)  0.95 (0.79–1.13)  1.00  0.89 (0.54–1.49) | Age, BMI, employment status, snoring, smoking, alcohol use, depressive symptoms, physical activity, coffee intake, multivitamin use, diabetes | 8 |
| Qian *et al*.,  2015, USA | F | Breast cancer | 1553 | 40013 | <6  6–<7  7–<8  8–<9  ≥9 | 0.87 (0.64–1.18)  1.04 (0.90–1.20)  0.93 (0.82–1.05)  1.00  1.00 (0.84–1.19) | Age, race, education, marital status, BMI, vigorous physical activity, smoking status, pack-year, year since quitting, age at first live birth, number of live birth, age at first birth, menopause, use of hormonal replacement therapy, use of multivitamin, history of diabetes, family of cancer, alcohol consumption | 8 |
| Khawaja *et al*.,  2014, USA | M | Lung cancer | 150 | 21026 | ≤6  7  ≥8 | 1.18 (0.77–1.82)  1.00  0.97 (0.67–1.41) | Age, race, parental history of cancer, exercise frequency, caloric intake, BMI, type 2 diabetes, alcohol consumption, smoking status, sleep apnea, snoring | 7 |
| Luojus *et al*.,  2014, Finland | M | Lung cancer | 81 | 2586 | ≤6.5  7–7.5  ≥8 | 2.01 (1.09–3.71)  1.00  1.96 (1.14–3.38) | Age, examination years, cumulative smoking history, family cancer history, human population laboratory depression scale scores, alcohol consumption, physical activity, BMI | 8 |
| Jiao *et al*., 2013,  USA | F | Colorectal cancer | 851 | 75828 | ≤5  6  7  8  ≥9 | 1.36 (1.06–1.74)  1.08 (0.91–1.28)  1.00  1.01 (0.84–1.21)  1.47 (1.10–1.96) | Age, ethnicity, fatigue, hormone replacement therapy, waist to hip ratio, physical activity | 7 |
| Wu *et al*., 2013,  Singapore | F | Breast cancer | 769 | 34028 | ≤6  7  8  ≥9 | 1.00  1.00 (0.84–1.19)  1.00 (0.84–1.21)  0.89 (0.64–1.22) | Age at recruitment, year of recruitment, dialect group, education, age when period became regular, parity, BMI and menopausal status | 8 |
| Luo *et al*., 2013,  USA | F | Thyroid cancer | 295 | 142933 | ≤6  7–8  ≥9 | 0.79 (0.61–1.02)  1.00  0.74 (0.39–1.39) | Age at enrollment, ethnicity, education level, smoking, BMI, recreational physical activity, alcohol intake, family history of cancer, previous thyroid disease, history of hormone therapy use, depression score, different treatment assignments for clinical trials | 7 |
| Vogtmann *et al*.,  2013, USA | F | Breast cancer | 5149 | 110011 | ≤5  6  7  8  ≥9 | 0.95 (0.85–1.07)  0.94 (0.87–1.00)  1.00  0.99 (0.92–1.06)  1.03 (0.90–1.18) | Age, clinical trial arm assignment, number of live births, age at menarche, age at menopause, BMI, energy expenditure, education, income, race/ethnicity, marital status, age at first births, previous use of hormone replacement therapy, history of benign breast disease, family history of breast cancer, alcohol consumption, smoking status | 8 |
| Zhang *et al*.,  2013, USA | M | Colorectal cancer | 709 | 30121 | ≤5  6  7  8  ≥9 | 0.67 (0.35–1.28)  1.14 (0.93–1.41)  1.00  1.22 (1.01–1.46)  1.35 (1.00–1.82) | Age, smoking before age 30, history of colorectal cancer in a parent or sibling, history of endoscopy, regular aspirin use, physical activity, snoring, BMI, history of diabetes, beef, pork, and lamb as a main dish, consumption of processed meat, alcohol consumption, energy-adjusted total calcium intake, total folate, Vitamin D intake | 7 |
|  | F | Colorectal cancer | 1264 | 76368 | ≤5  6  7  8  ≥9 | 1.10 (0.85–1.44)  1.04 (0.90–1.20)  1.00  1.16 (1.01–1.33)  1.11 (0.85–1.44) | Postmenopausal hormone use and factors listed in the above cell |  |
| Sturgeon *et al*.,  2012, USA | F | Endometrial cancer | 382 | 48725 | ≤6  7  8  ≥9 | 1.00  1.05 (0.83–1.34)  0.93 (0.71–1.23)  0.87 (0.51–1.46) | Age, race, body mass index, smoking, number of live births, physical activity, unopposed estrogen use, and family history of endometrial cancer | 8 |
| von Ruesten *et al*., 2012, Europe | M and F | All cancer | 846 | 23620 | <6  6–<7  7–<8  8–<9  ≥9 | 1.43 (1.09–1.87)  0.99 (0.82–1.20)  1.00  1.03 (0.87–1.23)  0.79 (0.61–1.04) | Age, sex, sleeping disorders, alcohol intake from beverages, smoking status, walking, cycling, sports, employment status, education, BMI, waist-to-hip ratio, prevalent hypertension at baseline, history of high blood lipid levels at baseline, consumption of caffeinated beverages, satisfaction with life, satisfaction with health, intake of antidepressants | 8 |
| Weiderpass *et al*.,  2012, Japan | F | Ovarian cancer | 86 | 45748 | <6  6–7  ＞7 | 1.00  0.5 (0.2–1.0)  0.4 (0.2–0.9) | Age at menarche, nulliparous, parity, age at first birth, breastfeeding, use of exogenous hormones, menopausal status at enrollment, height, body mass index, smoking status, exposure to second-hand smoke, physical activity during leisure time | 8 |
| Kakizaki *et al*.,  2008, Japan | M | Prostate cancer | 127 | 22320 | ≤6  7–8  ≥9 | 1.38 (0.77–2.48)  1.00  0.36 (0.18–0.72) | Age, marital status, education, job status, history of disease, family history of cancer, BMI, cigarette smoking, alcohol consumption, walking status | 8 |
| Kakizaki *et al*.,  2008, Japan | F | Breast cancer | 143 | 28515 | ≤6  7  8  ≥9 | 1.67 (1.00–2.78)  1.00  0.99 (0.59–1.65)  0.29 (0.09–0.98) | Age, BMI, history of disease, family history of cancer, job, marital status, education, cigarette smoking, alcohol consumption, time spent walking, total caloric intake, menopause status, age at menarche, age at first delivery, number of deliveries, using of oral contraceptive drugs, using of hormone drugs except for oral contraceptive drugs | 8 |
| Pinheiro *et al*.,  2006, USA | F | Breast cancer | 4223 | 77418 | ≤5  6  7  8  ≥9 | 0.93 (0.79–1.09)  0.98 (0.91–1.06)  1.00  1.05 (0.97–1.13)  0.95 (0.82–1.11) | Age, BMI, height, history of benign breast disease, family history of breast cancer, parity and age at first birth, age at menarche, age at menopause, postmenopausal hormone use, physical activity, alcohol and caloric intake, smoking | 6 |
| Verkasalo *et al*.,  2005, Finland | F | Breast cancer | 242 | 12222 | ≤6  7–8  ≥9 | 0.85 (0.54–1.34)  1.00  0.69 (0.45–1.06) | Age, zygosity, social class, number of children, use of oral contraceptives, BMI, alcohol use, smoking, physical activity | 8 |
| **Case-Control Study** | | | | | | | | |
| Xiao *et al*., 2016,  USA | F | Breast cancer | 519 | 42435 | <6  6  7  8  ≥9 | 1.09 (0.83–1.44)  1.01 (0.78–1.30)  1.19 (0.92–1.54)  1.00  1.08 (0.80–1.45) | Age, enrollment year, enrollment state, race, education, income, marital status, BMI, physical activity, overall sitting, smoking status, pack-year, number of live birth, age at first birth, length of breast feeding, age at menarche, menopause, use of menopausal hormone therapy, use of multivitamin, use of aspirin, history of diabetes, family history of cancer, alcohol consumption, dietary intake of total fat, fiber, folate, total calories | 8 |
| Wang *et al*.,  2015, China | F | Breast cancer | 654 | 1321 | ≤6  6.1–8.9  ≥9.0 | 1.53 (1.10–2.12)  1.00  1.59 (1.17–2.17) | Age, education, BMI, age at menarche, menopausal status, parity, physical activity, breast-feeding, family history of breast cancer, other sleep factors | 7 |
| Girschik *et al*.,  2013, Australia | F | Breast cancer | 1133 | 2828 | ＜6  6–7  7–8  ＞8 | 1.05 (0.82–1.33)  0.96 (0.80–1.16)  1.00  1.10 (0.87–1.39) | Age, number of children, age at first birth, breastfeeding, menopausal status, use of hormone replacement therapy, duration of use of hormone replacement therapy, alcohol consumption, comparative weight at age 30 years, ever use of melatonin, physical activity | 7 |
| McElroy *et al*.,  2006, USA | F | Breast cancer | 4010 | 9275 | <5  5.0–5.9  6.0–6.9  7.0–7.9  8.0–8.9  ≥9.0 | 0.94 (0.62–1.44)  0.82 (0.65–1.05)  0.89 (0.79–1.01)  1.00  1.01 (0.91–1.11)  1.01 (0.84–1.23) | Age, state, parity, age at first full-term pregnancy, family history of breast cancer, alcohol consumption, BMI, menopausal status, age at menopause, postmenopausal hormone use, education, marital status | 7 |

#: M: Males; F: Females;
