# Supplementary material for: Sleep duration and the risk of cancer: a systematic review and meta-analysis including dose–response relationship
Source: BMC Cancer. 2018 Nov 21;18:1149. doi: 10.1186/s12885-018-5025-y (PMC6249821; doi:10.1186/s12885-018-5025-y)
Supplement: Supplementary file 3 — Study quality of cohort studies included in the analysis of sleep duration and cancer risk. (DOCX 21 kb) [file 12885_2018_5025_MOESM3_ESM.docx]

**Additional file 3.** Study quality of cohort studies included in the analysis of sleep duration and cancer risk

| **Author, year** | **Study** | **Selection** | **Comparability** | **Outcome** | **Total Score** |
| --- | --- | --- | --- | --- | --- |
| Heckman *et al*., 2017 | Nurse’s Health Study Ⅱ | ** | ** | *** | 7 |
| Gu *et al*., 2016 | NIH-AARP Diet and Health Study Cohort | *** | ** | ** | 7 |
| Markt et al., 2016 | Health Professional Follow-Up Study | *** | ** | *** | 8 |
| Cohen et al., 2015 | Nurse’s Health StudyⅠ/Ⅱ  Health Professional Follow-Up Study | ** | ** | *** | 7 |
| Hurley et al., 2015 | California Teachers Study | ** | ** | ** | 6 |
| Markt et al., 2015 | Swedish National March  Cohort | *** | ** | *** | 8 |
| Qian et al., 2015 | Breast Cancer Detection  Demonstration Project | *** | ** | *** | 8 |
| Khawaja et al., 2014 | The Physicians’ Health Study | ** | ** | *** | 7 |
| Luojus et al., 2014 | The prospective cohort  Kuopio Ischemic Heart  Disease Study | *** | ** | *** | 8 |
| Jiao et al., 2013 | The Women’s Health  Initiative Observational Study | *** | ** | ** | 7 |
| Wu et al., 2013 | Singapore Chinese Health Study Cohort | *** | ** | *** | 8 |
| Luo et al., 2013 | The Women’s Health Initiative | *** | ** | *** | 8 |
| Vogtmann et al.2013 | The Women’s Health  Initiative | *** | ** | *** | 8 |
| Zhang et al., 2013 | The Health Professionals  Follow-up Study  The Nurses’ Health Study | ** | ** | *** | 7 |
| Sturgeon et al., 2012 | The Women’s Health  Initiative Observational Study | *** | ** | *** | 8 |
| von Ruesten et al., 2012 | The European Prospective  Investigation into Cancer and Nutrition | *** | ** | *** | 8 |
| Weiderpass et al., 2012 | The Japan Public Health  Center-based Prospective  Study | *** | ** | *** | 8 |
| Kakizaki et al., 2008  (Prostate Cancer) | The Ohsaki National Health Insurance Cohort Study | *** | ** | *** | 8 |
| Kakizaki et al., 2008  (Breast Cancer) | The Ohsaki National Health Insurance Cohort Study | *** | ** | *** | 8 |
| Pinheiro et al., 2006 | The Nurses’ Health Study | ** | ** | ** | 6 |
| Verkasalo et al., 2005 | The Finnish Twin Cohort | *** | ** | *** | 8 |
