# Supplementary material for: Sleep duration and the risk of cancer: a systematic review and meta-analysis including dose–response relationship
Source: BMC Cancer. 2018 Nov 21;18:1149. doi: 10.1186/s12885-018-5025-y (PMC6249821; doi:10.1186/s12885-018-5025-y)
Supplement: Supplementary file 4 — Study quality of case-control studies included in the analysis of sleep duration and cancer risk. (DOCX 20 kb) [file 12885_2018_5025_MOESM4_ESM.docx]

**Additional file 4.** Study quality of case-control studies included in the analysis of sleep duration and cancer risk

| **Author, publication year** | **Selection** | **Comparability** | **Exposure** | **Total score** |
| --- | --- | --- | --- | --- |
| Xiao et al., 2016 | *** | ** | *** | 8 |
| Wang et al., 2015 | ** | ** | *** | 7 |
| Girschik et al., 2013 | **** | ** | * | 7 |
| McElroy et al., 2006 | *** | ** | ** | 7 |
