# Supplementary figures and images for: Sleep duration and the risk of cancer: a systematic review and meta-analysis including dose–response relationship
Source: BMC Cancer. 2018 Nov 21;18:1149. doi: 10.1186/s12885-018-5025-y (PMC6249821; doi:10.1186/s12885-018-5025-y)

Meta-analysis estimates, given named study is omitted

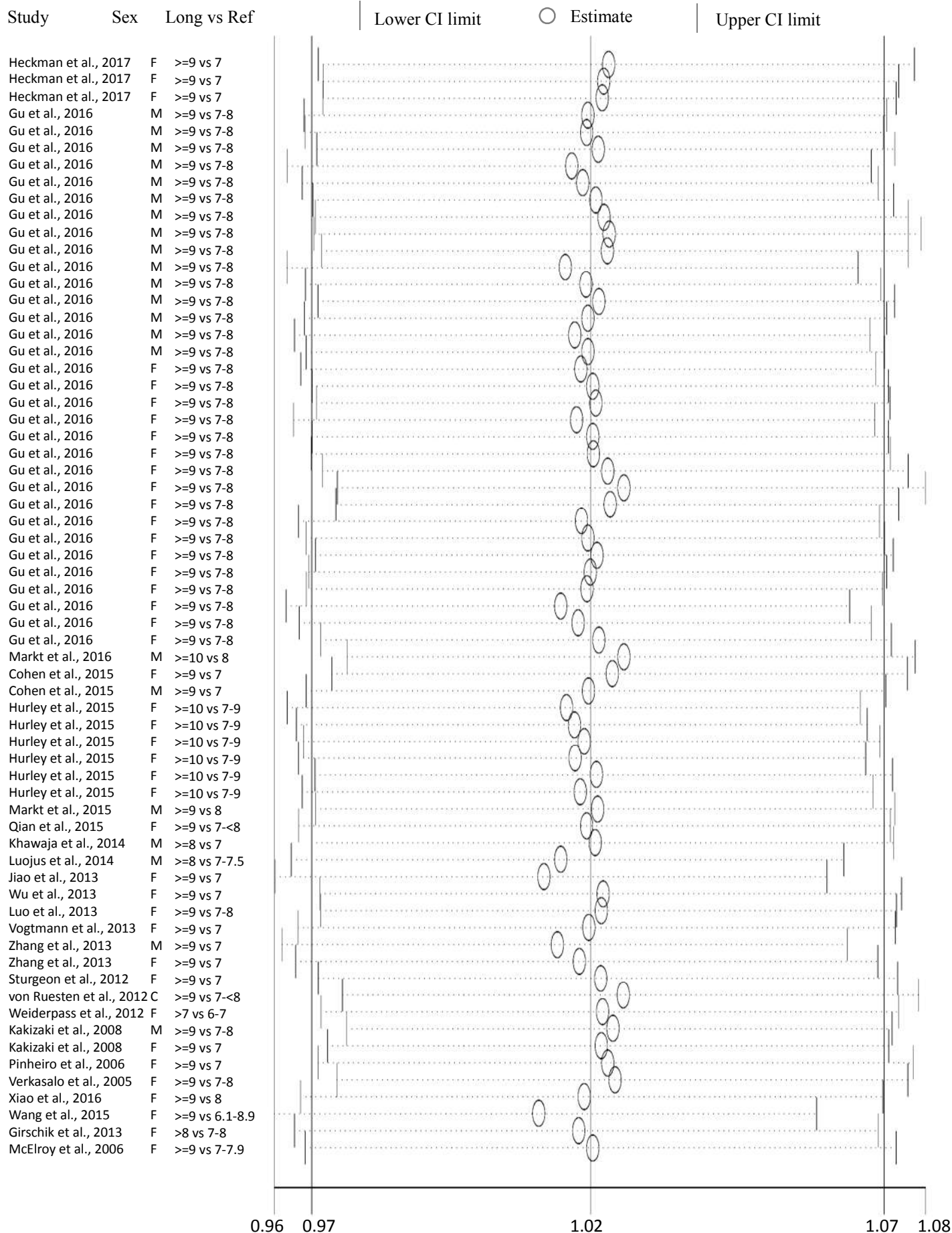

Supplement: Supplementary file 6 — Sensitivity analysis of sleep duration and cancer risk, longest vs. reference analysis. (PDF 186 kb) [file 12885_2018_5025_MOESM6_ESM.pdf]

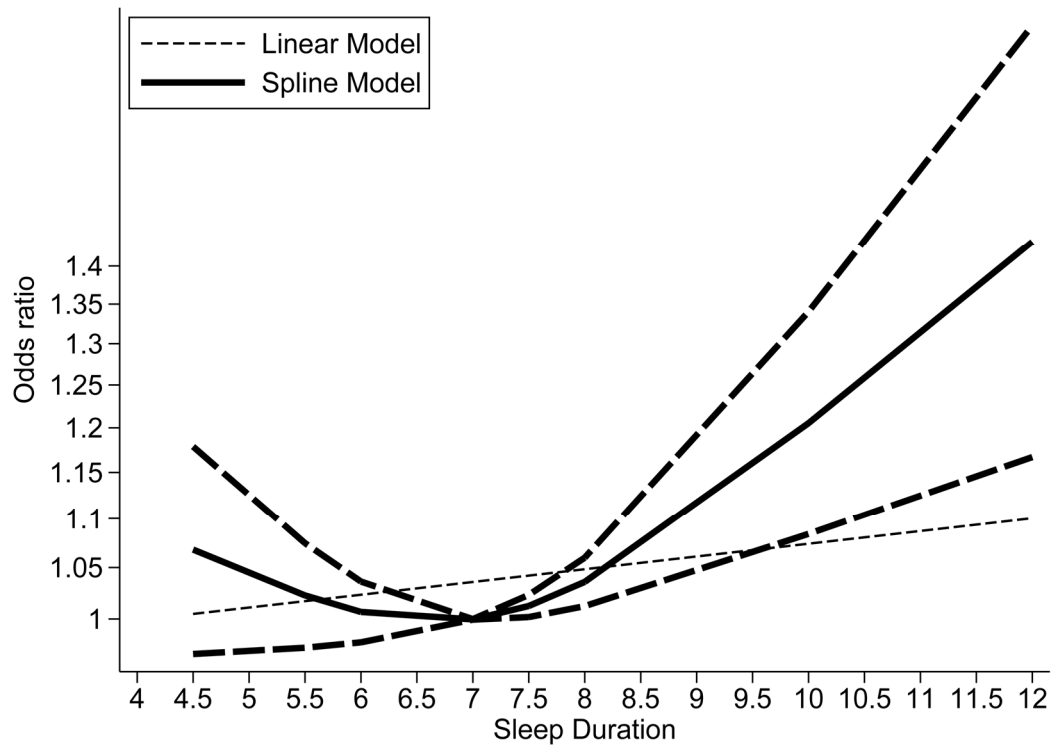

Supplement: Supplementary file 8 — Nonlinear dose–response analyses of sleep duration and colorectal cancer risk. (PDF 79 kb) [file 12885_2018_5025_MOESM8_ESM.pdf]
