# Supplementary material for: Sleep duration and the risk of cancer: a systematic review and meta-analysis including dose–response relationship
Source: BMC Cancer. 2018 Nov 21;18:1149. doi: 10.1186/s12885-018-5025-y (PMC6249821; doi:10.1186/s12885-018-5025-y)
Supplement: Supplementary file 7 — Association between sleep duration and cancer risk from nonlinear dose–response analysis. (DOCX 20 kb) [file 12885_2018_5025_MOESM7_ESM.docx]

**Additional file 7.** Association between sleep duration and cancer risk from non-linear dose-response analysis

| **Sleep duration** | **No of Articles/Studies** | **Cancer risk** |
| --- | --- | --- |
| 4.5 | 8/45 | 1.017 (0.981–1.054) |
| 5.5 | 15/49 | 1.006 (0.989–1.024) |
| 6 | 7/8 | 1.003 (0.993–1.013) |
| 6.5 | 4/4 | 1.000 (0.996–1.005) |
| 7 | 13/17 | 1.00 |
| 7.5 | 8/39 | 1.002 (0.997–1.007) |
| 8 | 14/23 | 1.007 (0.993–1.021) |
| 8.5 | 3/3 | 1.014 (0.988–1.041) |
| 9 | 3/3 | 1.022 (0.982–1.065) |
| 10 | 16/52 | 1.044 (0.968–1.125) |
